# The pelvic area — A central hypogastric area for abdominal palpation for women with abdominal pain: A narrative review

**DOI:** 10.1016/j.amsu.2022.104000

**Published:** 2022-06-14

**Authors:** Ming Cheh Ou, Dennis Ou, Chung Chu Pang

**Affiliations:** a Department of Obstetrics and Gynecology, Zhong-Xiao Branch, Taipei City Hospital, Taipei City, Taiwan; b Department of Mechanical Engineering and Biomedical Engineering, Carnegie Mellon University, Pittsburgh, USA; c Department of Occupational Therapy, National Taiwan University, Taipei City, Taiwan

**Keywords:** Surface anatomy, Pelvic area, Palpation, abdominal pain, Ou MC manipulation, Ou MC decrescendo Phenomenon, APOM, Abdominal palpation with Ou MC manipulation, OuDP, Ou MC decrescendo phenomenon, Ou MC, Ou, Ming Cheh

## Abstract

The pelvic area is a central hypogastric area which is conformed with the pelvic inlet to reduce overlapping of the non-pelvic area and is more sensitive and specific in diagnosing female abdominal emergencies than the traditional four quadrants and nine regions methods for abdominal palpation. The purpose of this narrative review is to assess the principle and mechanism of formation of the pelvic area for abdominal palpation for women with abdominal pain. By classifying the abdominal area as inside or outside the pelvic area, abdominal pain can be located inside or outside the pelvic cavity, thereby distinguishing pelvic diseases from non-pelvic diseases. When the examiner divides the patient's pelvic area along the pelvic ring using his/her hand on the patient's contralateral abdomen, there can be a reduced pain zone under the hand and alleviate pain in the non-diseased area. It allows patients with poor perception of tenderness or abdominal pain with guarding to easily recognize pelvic or non-pelvic pain. Partitioning the pelvic area which conforms with pelvic cavity inlet can reduce confusing pelvic and non-pelvic diseases when using traditional four quadrants or nine regions method. The division of the pelvic area on the patient's contralateral side can induce a reduced pain zone under the hand and alleviate pain in the non-diseased area, which can help the patient distinguish between pelvic and non-pelvic pain. Pain is a subjective feeling to the patient, and correct patient perception of pain are the basis for a correct diagnosis.

## Highlights

• Pelvic area conforming with pelvic inlet for abdominal palpation reduces confusing pelvic and non-pelvic diseases.
• Ou MC decrescendo phenomenon (OuDP) secluding the pelvis makes pelvic area wieldy for abdominal pain location.
• OuDP by the examiner’s hand on the patient’s contralateral abdomen reduces pain under the hand and the non-diseased area.
• Pelvic area shows high sensitivity and specificity for locating pelvic and non-pelvic pain, thereby diseases.

## 2 Introduction

**Fig. 1:**
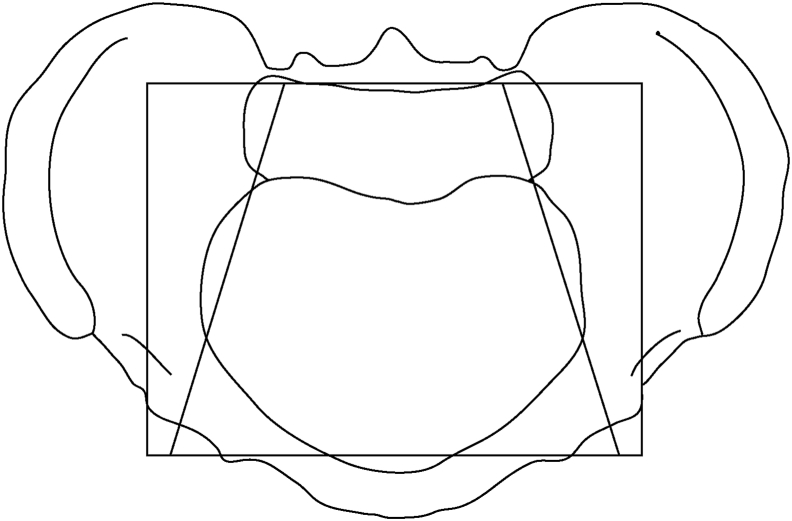
The pelvic area (Ou MC area) traces along the pelvic ring, containing most of the pelvic organs. Thus, this trapezoid isolated anatomical functional area is more closely related to the functions of the organs in the pelvic cavity than the traditional hypogastric region. The square hypogastric region may involve parts of non-pelvic organs, as its shape does not conform to the pelvic inlet (5).

The traditional abdominal palpation method usually divides the abdomen into four quadrants or nine regions and does not identify the position of the pelvic cavity for diagnosis [1,2]. Even the hypogastric region of the nine regions method which is on the pelvis does not conform to the pelvic cavity, making it difficult to distinguish between pelvic and non-pelvic diseases in clinical practice (Fig. 1) [1].

In women, the organs located in the pelvic cavity include the uterus, ovaries, fallopian tubes, bladder, sigmoid colon, and rectum. Inflammation of the female reproductive system often causes pain in the pelvic cavity. The resulting pain often overlaps with pain in other parts of the abdomen, making the diagnosis difficult [1,2]. Therefore, it is important to determine the position of the pelvic cavity during palpation to help distinguish between pelvic and non-pelvic diseases [3,4]. This review paper aims to depict the principles and mechanism of delimiting of the pelvic area which is a surface anatomical area conforms to the pelvic cavity for abdominal palpation to distinguish between pelvic and non-pelvic pain (Fig. 1) [[3], [4], [5]].

## 3 Abdominal palpation for women with abdominal pain

### 3.1 Traditional abdominal palpation with four quadrants and 9 regions methods

The abdominal four-quadrant method does not delimit the pelvic cavity area of the abdomen and the pelvic cavity is divided into left and right sides with the vertical median plane through the umbilicus. The left and right sides of the pelvic cavity merge with other abdominal areas on both sides, which makes it easy to confuse pelvic diseases with other abdominal diseases. For example, in the right lower abdominal area, pain may be caused by diseases of the appendix, caecum, colon, kidney, or pelvic organs [2]. Fig. 1 shows the hypogastric region is square in shape, boundaries with intertubercular and vertical midclavicular planes and contains part of the non-pelvic cavity area, which also makes it difficult to distinguish between pelvic and non-pelvic diseases [1,5]. Thus, when distinguishing between pelvic cavity disease and non-pelvic cavity disease, the traditional abdominal division method is often confusing.

### 3.2 Abdominal palpation with the pelvic area conformed with pelvic ring

The pelvic area is conformed with the pelvic inlet in the shape of a trapezoid with the shorter parallel formed by the upper part of the sacrum. Both sides are along the external iliac artery and the longer parallel is formed by the pubic bone (Fig. 1). If there is pain in the pelvic area, diseases that cause inflammation of the caecum, stomach, and large intestine can be ruled out. If there is no pain in the pelvic area, diseases of the reproductive system, bladder, and rectum can be ruled out [[4], [5], [6]].

Ou MC (Ou, Ming Cheh) manipulation is a method developed by Ou MC et al. to divide the abdominal surface into the pelvic and non-pelvic areas according to the position of pelvic cavity around the pelvic ring to easily distinguish between pelvic and non-pelvic pain, thereby distinguish pelvic diseases from non-pelvic diseases [4,5].

## 4 Delimitation of the pelvic area by Ou MC manipulation for clinical practice

**Fig. 2:**
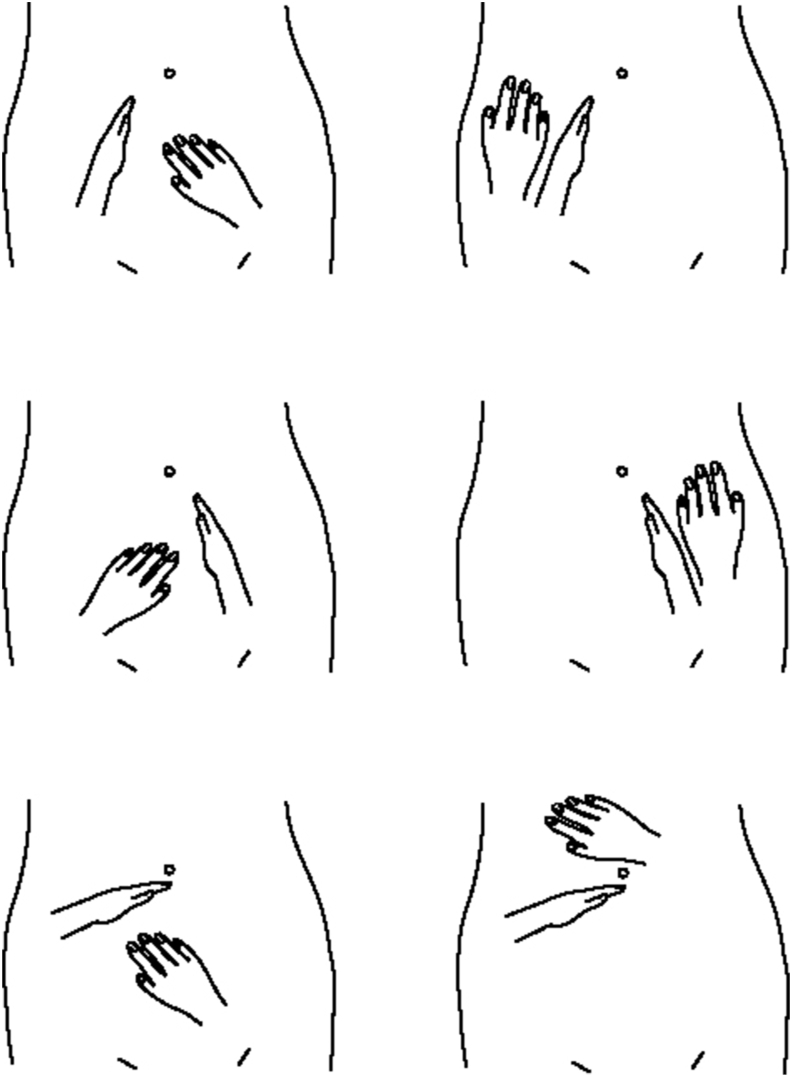
Abdominal palpation with Ou MC manipulation. The edge of the examiner's hand is placed on the patient's contralateral side abdomen along a line from the subumbilicus to the femoral arterial canal of the inguinal area with adequate pressure against the pelvic wall; the other hand is used to palpate either side. Finally, a hand is placed contralateral to the patient's side on top of the pelvic area (Ou MC area), and either side of the separating hand is palpated using the other hand (Modified from Ref. 5).

**Fig. 3:**
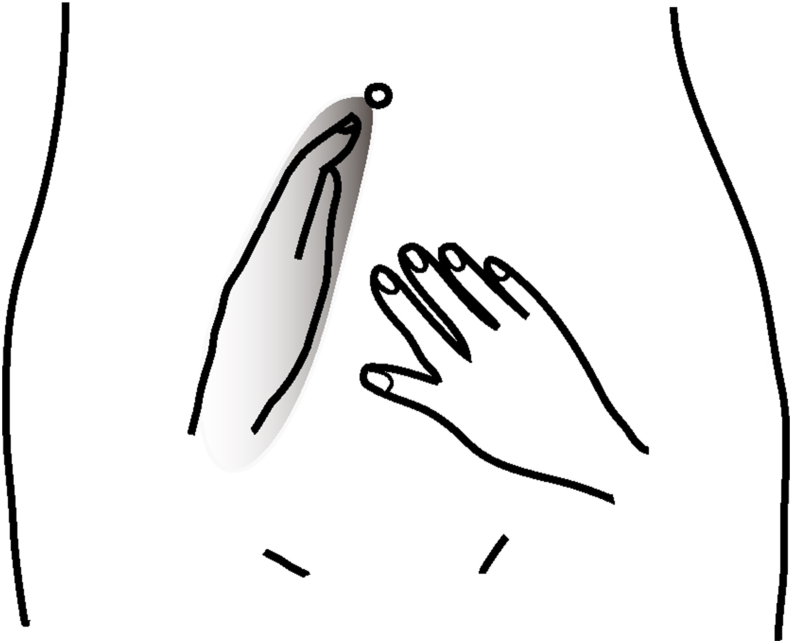
The examiner puts the hand on the patient's contralateral side abdomen for 5 seconds or more or repeats the procedure. It can produce a pain-reduced area under the hand and alleviate the pain in the non-diseased area, which allows patients with abdominal pain and guarding, or poor perception of tenderness to easily recognize pelvic or non-pelvic pain (5). By bending the upper end of the finger, the upper abdomen and the pelvic cavity area can be separated simultaneously, while examining the left or right abdomen.

Abdominal palpation with Ou MC manipulation (APOM) was performed by placing a hand in a chopping gesture contralateral to the patient's side along a line from the subumbilicus to the femoral arterial canal of the inguinal area while the patients were in the lithotomy or supine position with abdominal muscle relaxed (Fig. 2, Fig. 3) [4,5]. Adequate pressure is applied against the pelvic wall to separate the pelvic area from other abdominal areas. The other hand is used to palpate either side of the separating hand. Finally, the separating hand is placed contralateral to the patient's side horizontally on the top of the pelvic area, and either side of the separating hand are palpated using the other hand (Fig. 2) [5]. By bending the upper end of the finger, the upper abdomen and the pelvic cavity area can be separated simultaneously, while examining the left or right abdomen (Fig. 3).

In patients with abdominal muscle guarding or poor perception of tenderness, the separating hand is placed for 5 seconds or more or repeats the procedure and does not need to be pressed heavily. It will produce a pain-reduced area under the hand and alleviates the pain in the non-diseased area (Fig. 3), which allows patients to easily recognize whether it is pelvic or non-pelvic pain and is called Ou MC decrescendo phenomenon (OuDP) [5].

When the pain in the pelvic cavity is too weak or too deep for the patient to confirm, diseases outside the pelvic cavity can be excluded by APOM first, followed by a vaginal examination to confirm weak or deep pelvic pain. Additionally, vaginal examination assisted with Ou MC manipulation can make it easier for the patient to recognize whether it is pelvic or non-pelvic pain. With OuDP induced by Ou MC manipulation, pelvic pain in vaginal examination remains with pelvic diseases, but vanishes or alleviates with non-pelvic diseases as does in APOM [4,5].

**Fig. 4:**
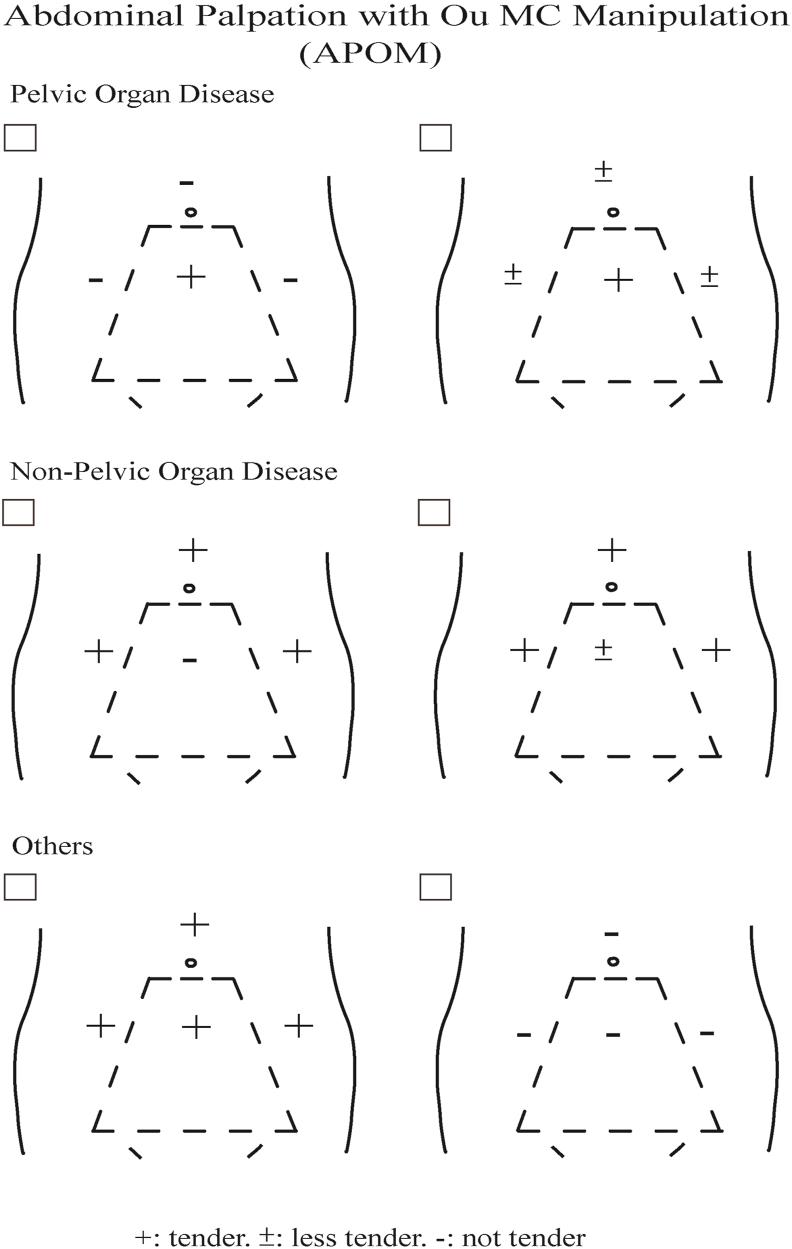
The chart for abdominal palpation with Ou MC manipulation. If the tenderness is more prominent or located in the pelvic area (the trapezoidal sub-abdominal area defined by Ou MC manipulation), pelvic organ disease is suspected. On the contrary, the opposite test result excludes pelvic organ diseases. Significant tenderness on both sides indicates disease on either side or the inflammation caused by a serious disease spread to the other side (5).

## 5 Interpretations for APOM (Fig. 4) [5]

1. If there is local tenderness in the pelvic area and there is no obvious tenderness in the non-pelvic area; the pain is caused by disease of the pelvic cavity.
2. If there is local tenderness in the non-pelvic area, but there is no obvious tenderness in the pelvic cavity area; the pain is caused by non-pelvic disease.
3. In a few cases, there is obvious tenderness in both areas, which means that there is disease on both sides or the inflammation caused by the disease of one side spreading to the other side.
4. Tenderness under the hand while separating the pelvic area is not suitable for diagnosis using this method, because it may be the result of compression of large blood vessels.

## 6 Clinical study for abdominal palpation with Ou MC manipulation (APOM)

A pilot prospective crossover study was initiated by Ou MC et al., in 2006 for evaluation of a cohort of 68 women with acute abdomen and a final diagnosis of pelvic inflammatory diseases (PID) with abdominal palpation with APOM and bimanual vaginal examination [4]. All the 68 women exhibited pelvic area tenderness with APOM. The bimanual pelvic examination indicated PID in 61 of these patients (90%) but exhibited no pelvic organ tenderness in 7 patients. Thus, APOM yielded greater sensitivity for PID than did the bimanual pelvic examination (P = 0.013). In the study, 6 patients exhibited no pelvic organ tenderness on bimanual pelvic examination. All of them had fluid-filled tubes or tuboovarian complex in the adnexal area, which might not be easily accessible on bimanual pelvic examination, whereas APOM can approach the abdominal area over adnexae effortlessly. Thus, APOM was able to locate diseased pelvic organs not easily assessed by bimanual pelvic examination. Bimanual pelvic examination appears to be a limited screening test for conditions affecting the female upper genital tract, even under the best circumstances [7].

In another prospective study with crossover design carried out from 2006 to 2009 by Ou MC et al. [5], a total of 259 consecutive women with acute abdomen presented to the emergency department and underwent traditional abdominal palpation and APOM. One hundred and thirteen patients had a final confirmed diagnosis. All the patients were in the lithotomy position. Of the 113 women with final confirmed diagnosis, 91 had pelvic organ diseases, 21 had non-pelvic organ diseases, and one had both pelvic and non-pelvic organ diseases. The results showed that the sensitivity of APOM for the diagnosis of pelvic organ diseases was significantly higher than that of traditional palpation (P = 0.003). APOM also showed a higher specificity than traditional palpation for excluding pelvic organ diseases (P = 0.003). Overall, 42 patients (37.2%) with abdominal guarding had to repeat the operation or aggravate separation in APOM to identify the painful area, which induced a pain-reduced area under the separating hand and alleviated the pain in non-diseased areas, making the tenderness more distinct in the area of diseased organ to be easier for the patient to identify. This pain relief is called the Ou MC decrescendo phenomenon (OuDP) in the study [5].

The other single blinded self-controlled study carried out from 2010 to 2011 by Ou MC et al. [6,8,9] for that APOM did not induce OuDP while using a hand ipsilateral to the patient's side to separate. The study compared the effect of either hand of the examiner with patient blinded on the contralateral or ipsilateral side of the patient's abdomen when used to alleviate acute abdomen pain in 39 women in lithotomy position. In this case, usage of the contralateral hand alleviated the pain of 92.3% of women (36/39), while the ipsilateral hand did not (0/39) (P < 0.001, Paired-t test).

## 7 Principle and mechanism for delimitation of the pelvic area

### 7.1 The delimitation of the pelvic area

The pelvic area is separated along the pelvic ring, involving fewer non-pelvic cavity areas than the hypogastric region. Therefore, compared to the traditional hypogastric region, the pelvic area is more precise for palpation to distinguish between pelvic pain and non-pelvic pain for its trapezoidal shape conforming with the pelvic inlet (Fig. 1) [3,5]. Furthermore, APOM classifies the abdominal area as inside or outside the pelvic area; thus, the diseased organ can be located inside or outside the pelvic cavity, which can reduce uncertainty. Therefore, APOM helps to identify the location of tenderness by using a partition method that reduces overlap with the pelvic cavity.

In the study carried out from 2006 to 2009 by Ou MC et al. [5], pelvic cavity shielding and organ isolation by the separating hand for the patients with abdominal guarding was found to assist in determining the location of tenderness by APOM suggesting that a wall-off effect of tenderness might occur. Nonetheless, visceral pain afferent to the pelvic organs is also innervated by the appendix, ureter, and colon, which indicates that there may be factors other than visceral afferents

### 7.2 Ou MC decrescendo phenomenon (OuDP)

Studies by Ou MC et al. from 2012 to 2021 on assessing OuDP have shown that it may directly normalize the dysfunctional tissue and result in an anti-inflammatory effect because OuDP renders it too fast to be considered an indirect effect [6,[8], [9], [10], [11], [12]]. Patients touch or press the pain area by themselves with the opposite hand, and the pain or inflammation is reduced, which also indicates the tissue function is restored by OuDP [6,[8], [9], [10], [11], [12], [13], [14], [15]].

The effect of OuDP (Fig. 3) may be responsible for the wall-off consequence by pelvic cavity shielding and organ isolation with the separating hand for the patients with abdominal guarding examined with APOM in the study from 2006 to 2009 by Ou MC et al. [5].

## 8 Limitations for abdominal palpation with Ou MC manipulation (APOM)

It is understandable that if the patient's abdominal cavity has both pelvic and non-pelvic organ diseases or if complications create tenderness in another area, APOM may not be able to detect the location of the diseased organ. In addition, a woman's pelvic area mainly constitutes reproductive organs, bladder, and large intestine. If pelvic organs are removed by surgery, other diseased organs may fill the pelvis and cause significant tenderness in the pelvic cavity. APOM is an abdominal palpation technique. If the patient is incapable of communicating with the examiner regarding the perception of pain, it cannot accurately detect the location of the pain.

## 9 Clinical implications

The proximity of intra-abdominal organs can cause significant overlap of abdominal pain presentation and the visceral pain is difficult to locate for a low density of visceral sensory innervation and extensive divergence of visceral input within the CNS, which makes the delimitation of the abdominal surface area for pelvic cavity is unwieldy and infeasible.[16] Nonetheless, pelvic pain is a common presentation for women and the high frequency of gynecologic diseases in women of childbearing age further complicates the differentiation of the sources of abdominal pain that the delimitation of the pelvic cavity area for abdominal palpation is essential. OuDP to seclude the pelvic cavity from other abdominal cavity makes the delimitation of pelvic cavity with Ou MC manipulation feasible. Peter Rosen [2] in 2011 commented Ou MC manipulation as a useful method for diagnosis of abdominal pain [4]. Ou MC manipulation for locating pelvic and non-pelvic pain shows high sensitivity and specificity. Evaluation for abdominal pain can be challenging because of a broad differential diagnosis and because many associated signs and symptoms are nonspecific and further studies are warranted.

## 10 Conclusions

Partitioning the pelvic area which conforms with pelvic cavity inlet can reduce confusing pelvic and non-pelvic diseases when using traditional four quadrants or nine regions method. By classifying the abdominal area as inside or outside the pelvic area, abdominal pain can be located inside or outside the pelvic cavity, thereby distinguishing pelvic diseases from non-pelvic diseases. The division of the pelvic area on the patient's contralateral side with Ou MC manipulation can induce a reduced pain zone under the hand and alleviate pain in the non-diseased area, which can help the patient distinguish between pelvic and non-pelvic pain. Pain is a subjective feeling to the patient. Good patient communication and correct patient perception of pain are still the basis of a correct diagnosis.

## Ethical approval

The authors declare no ethical conflict to disclose.

## Sources of funding for your research

This research received no specific grant from any funding agency in the public, commercial, or not-for-profit sectors.

## Author contribution

All authors have made substantial contributions to conception and design, or acquisition of data, or analysis and interpretation of data, been involved in drafting the manuscript or revising it critically for important intellectual content, given final approval of the version to be published.

## Registration of research studies

All studies in the review are in compliance with the register rule of Taiwan which is based on ISRCTN.

## Consent

All studies in the review had consents of the patients obtained.

## Guarantor

All authors are in full responsibility for the work, the conduct of the study and had access to the data, and controlled the decision to publish.

## Provenance and peer review

Not commissioned, externally peer reviewed.

## Declaration of competing interest

The authors declare no conflicts of interests to disclose.
